# Anti-glypican-1 antibody–drug conjugate is a potential therapy against pancreatic cancer

**DOI:** 10.1038/s41416-020-0781-2

**Published:** 2020-03-10

**Authors:** Takahiko Nishigaki, Tsuyoshi Takahashi, Satoshi Serada, Minoru Fujimoto, Tomoharu Ohkawara, Hisashi Hara, Takahito Sugase, Toru Otsuru, Yurina Saito, Shigehiro Tsujii, Taisei Nomura, Koji Tanaka, Yasuhiro Miyazaki, Tomoki Makino, Yukinori Kurokawa, Kiyokazu Nakajima, Hidetoshi Eguchi, Makoto Yamasaki, Masaki Mori, Yuichiro Doki, Tetsuji Naka

**Affiliations:** 1 Department of Gastroenterological Surgery, Osaka University Graduate School of Medicine, Suita, Japan; 2 Laboratory of Immune Signal, National Institute of Biomedical Innovation, Health and Nutrition, Ibaraki, Japan; 3 Center for Intractable Immune Disease, Kochi University, Nankoku, Japan; 4 Department of Surgery, Kochi Medical School, Nankoku, Japan; 5 Animal Models of Human Diseases, National Institute of Biomedical Innovation, Health and Nutrition, Ibaraki, Japan; 6 Department of Surgery and Science, Graduate School of Medical Sciences, Kyusyu University, Fukuoka, Japan

**Keywords:** Pancreatic cancer, Chemotherapy

## Abstract

**Background:**

Pancreatic cancer (PDAC) is the most lethal malignancy. New treatment options for it are urgently required. The aim was to develop an antibody–drug conjugate (ADC) targeting glypican-1 (GPC-1) as a new therapy for PDAC.

**Methods:**

We evaluated GPC-1 expression in resected PDAC specimens and PDAC cell lines. We then measured the antitumour effect of anti-GPC-1 monoclonal antibody conjugated with the cytotoxic agent monomethyl auristatin F (MMAF) in vitro and in vivo.

**Results:**

GPC-1 was overexpressed in most primary PDAC cells and tissues. The PDAC cell lines BxPC-3 and T3M-4 strongly expressed GPC-1 relative to SUIT-2 cells. Compared with control ADC, GPC-1-ADC showed a potent antitumour effect against BxPC-3 and T3M-4, but little activity against SUIT-2 cells. In the xenograft and patient-derived tumour models, GPC-1-ADC significantly and potently inhibited tumour growth in a dose-dependent manner. GPC-1-ADC-mediated G2/M-phase cell cycle arrest was detected in the tumour tissues of GPC-1-ADC-treated mice relative to those of control-ADC-treated mice.

**Conclusions:**

GPC-1-ADC showed significant tumour growth inhibition against GPC-1-positive pancreatic cell lines and patient-derived, GPC-1-positive pancreatic cancer tissues. Our preclinical data demonstrated that targeting GPC-1 with ADC is a promising therapy for patients with GPC-1-positive pancreatic cancer.

## Background

Despite the trend towards increasing cancer survival, the prognosis for pancreatic ductal adenocarcinoma (PDAC) remains poor. PDAC is currently the fourth leading cause of cancer-related mortality. However, it is predicted to ascend to the second place in Western countries by 2030.^1^ PDAC is asymptomatic until the sudden onset of prominent clinical symptoms and advanced disease. Surgical resection is most likely to affect a cure. Surgical resection and adjuvant chemotherapy improve the prognosis of pancreatic cancer, and the overall median survival reaches 3–4 years after resection.^2–4^ On the other hand, about 80% of all patients are unresectable when they were discovered. The median survival for these patients with unresectable metastatic PDAC is < 1 year, and the 5-year overall survival rate is only 6%.^5^ Although several clinical trials have reported to improve the prognosis of metastatic PDAC, the clinical outcome for patients with metastatic PDAC remains poor.^6,7^ There is, therefore, an urgent need for new, efficacious approaches for PDAC treatment.

Antibody–drug conjugates (ADC) improve the therapeutic indices of cytotoxic anticancer agents. This approach uses immunoconjugates that are cytotoxic agents chemically or enzymatically linked to an antibody selectively binding internalising tumour-associated antigens.^8,9^ This strategy delivers the cytotoxic agent to the tumour site whilst minimising healthy tissue exposure. Recently ADC development has changed dramatically since the approval of Adcetris^®^ (brentuximab vedotin) in 2011 for the treatment of CD30-positive lymphomas,^10,11^ Kadcyla^®^ (ado-trastuzumab emtansine) in 2013 for the treatment of HER2-positive breast cancer^12–14^ and Besponza^TM^ (Inotuzumab Ozogamicin) in 2017 for the treatment of relapsed/refractory B-cell precursor acute lymphoblastic leukaemia.^15,16^ These successes bolstered ADC development. Fifty ADCs are in the pipeline for the treatment of haematological and solid tumour malignancies. A critical consideration in ADC design is the target choice as it substantially contributes to antitumour activity and ADC tolerability. ADC targets may occur on tumour cells, on tumour-associated cells such as tumour endothelial cells or in the tumour microenvironment. The target antigen should express on the surfaces of tumour rather than normal cells. Moreover, for differential cancer cell expression, antibody–drug conjugate targets must have extracellular epitopes that bind specific antibodies and internalise in the target cells where the drug should be released.

Glypican-1 (GPC-1) is a heparan sulfate proteoglycan (HSPG) that binds to the plasma membrane by a glycosyl-phosphatidylinositol (GPI) anchor.^17,18^ We identified GPC-1 as an antigen for oesophageal squamous cell carcinoma (ESCC) using quantitative proteomics targeting the cell surface membrane protein. GPC-1 expression was very weak or undetectable in the heart, kidney, ovary, placenta, adrenal gland, thyroid, lung, liver, pancreas, stomach, small intestine, colon, prostate, thymus and brain.^19,20^ Thus, GPC-1 is a promising target for ESCC. It has recently been reported that GPC-1 was expressed in PDAC.^21,22^

We produced a new ADC system using anti-GPC-1 monoclonal antibody and monomethyl auristatin F (MMAF), and demonstrated its potential effectiveness against uterine cervical cancer.^23^ The aims of this study were to investigate the GPC-1 expression in pancreatic cancer, and assess the feasibility of applying GPC-1-ADC as a new drug delivery technology.

## Materials and methods

### Patients and biopsy materials

Pancreatic cancer tissue was obtained from 75 patients who underwent R0 pancreatectomy at the Department of Gastroenterological Surgery, Osaka University Hospital, between 2008 and 2012. Informed consent was obtained from all donors. All studies involving human subjects were approved by the Institutional Review Board (No. 15478-4) of Osaka University Hospital and by the National Institute of Biomedical Innovation, Health and Nutrition (No. 94). Diagnoses of all tumours as pancreatic cancer were confirmed following histological review by board-certified pathologists. TNM 7th edition (Union for International Cancer Control (UICC)) criteria were used to categorise pathological staging.

### Immunohistochemistry

Three-micrometre sections were prepared from formalin-fixed, paraffin-embedded tissue samples. As described previously,^19^ the sections were deparaffinised with xylene and rehydrated in four graded alcohol solutions (70%, 80%, 90% and 100%). Immunohistochemical (IHC) staining for GPC-1 was performed using rabbit polyclonal anti-GPC-1 antibody (Atlas Antibodies AB, Stockholm, Sweden, 1:400) and visualised with Envision ChemMate (Dako, Glostrup, Denmark) according to the manufacturer’s protocol.

Immunostaining was scored according to the intensity of the staining: 0, no staining; 1, normal staining; 2, strong staining. The ‘density’ of staining (termed the positivity score) was as follows: 1, indicates less than 50% positivity; 2, indicates more than 50% positivity. The final IHC score was determined by multiplying the intensity score by the positivity score, resulting in a maximum possible score of 4. These data were referred to as the GPC-1 score. Furthermore, we divided patients into two equally balanced groups by score.

### Cell lines and culture

BxPC-3 cells were obtained from the European Collection of Authenticated Cell Cultures (ECACC, Salisbury, UK). SUIT-2 cells were acquired from the Japanese Collection of Research Bioresources (Osaka, Japan). T3M-4 cells were procured from the RIKEN BioResource Center (Wako, Japan). BxPC-3 and SUIT-2 cells were maintained in RPMI 1640 medium. T3M-4 cells were maintained in Ham’s F-10 medium. All media were supplemented with 10% foetal bovine serum (FBS, Serum Source International, Charlotte, NC, USA) and 100 U mL^−1^ penicillin + 100 µg mL^−1^ streptomycin (Nacalai Tesque Inc., Kyoto, Japan). Cultures were maintained at 37 °C under a humidified atmosphere at 5% CO_2_.

### Antibody generation

To generate monoclonal antibodies (mAbs) against human GPC-1, 4–6-week mice with MRL or C3H backgrounds were immunised with recombinant human GPC-1 protein (R&D Systems, Minneapolis, MN, USA) as previously reported.^23^

### Preparation of antibody–drug conjugate

As described previously,^23^ the GPC-1 mAb (clone 01a033) and isotype control antibody (mouse IgG2a, clone MOPC-173, Biolegend, San Diego, CA, USA) were used to synthesise the ADC. The GPC-1 mAb was partially reduced with tris-(2-carboxyethylphosphine) hydrochloride (TCEP) followed by reaction with maleimidolcaproyl–valine–citrulline–*p*-aminobenzyloxycarbonyl–monomethyl auristatin F (mc–vc–PABC–MMAF)^24^ to yield GPC-1-ADC and control ADC, respectively. To remove residual unreactive toxins, the conjugated ADCs were desalted on Sephadex G50 columns, and the buffer was replaced with phosphate-buffered saline (PBS) and filtered. The drug-to-antibody ratio (DAR) was determined from the ratio of A248 nm:A280 nm. The DAR was 4.1 for GPC-1-ADC and 3.8 for control ADC. The drug distribution was analysed by hydrophobic interaction chromatography (HIC).

### Quantitative flow cytometric analysis

Pancreatic cancer cells were grown to 80% confluency in 100-mm dishes. As described previously,^20^ cells were washed twice in PBS (Nacalai Tesque Inc., Kyoto, Japan) and detached in 0.02% (w/v) EDTA (Nacalai Tesque Inc., Kyoto, Japan). Cells were washed twice with cold FACS buffer (PBS supplemented with 1% (w/v) FBS and 0.1% (w/v) sodium azide), then incubated with mouse anti-GPC-1 antibody (clone 01a033) at 10 µg mL^−1^ and labelled with FITC-labelled goat anti-mouse IgG (H + L chain-specific) antibody (Southern Biotech, Birmingham, AL, USA). Stained cells were viewed under a FACS Canto II cytometer (Becton Dickinson, Mountain View, CA, USA) and analysed with FlowJo software (Tree Star, Stanford, CA, USA).

As described previously,^23^ plasma membrane GPC-1 expression levels were quantified by QIFIKIT flow cytometric indirect immunofluorescence assay (Dako, Hamburg, Germany) using anti-GPC-1 mAb (clone 01a033) as the primary antibody. Per sample, 10^5^ cells were incubated in a saturating concentration (10 µg mL^−1^) of primary antibody for 30 min at 4 °C. After washing, FITC-conjugated secondary antibody (1:50) was added for 45 min at 4 °C. Antibody binding was measured in a FACS Canto II cytometer (Becton Dickinson, Mountain View, CA, USA). The specific antigen density was calculated by subtracting the background antibody equivalent from the antibody-binding capacity based on a standard curve of log mean fluorescence intensity against log antibody-binding capacity.

### Cell proliferation assay

Pancreatic cancer cells were plated in 96-well plates at a density of 1500 cells well^−1^ (90 µL well^−1^) and incubated at 37 °C under a 5% CO_2_ atmosphere. After overnight incubation, various concentrations of MMAF (0–1000 nM), anti-GPC-1 antibody 01a033 (0–16 nM), GPC-1-ADC (0–16 nM) and control ADC (0–16 nM) were added. Cell viability was assessed after 144 h using a Cell Titer-Glo luminescent assay kit (Promega, Madison, WI, USA) according to the manufacturer’s instructions. Percentage survival was calculated by dividing the measured luminescence per drug or ADC concentration by the mean number of untreated cells (in growth medium), and multiplying the quotient by 100. The IC_50_ was then calculated.

### Internalisation studies

BxPC-3 and T3M-4 cells (10^6^ tube^−1^) were incubated for 1 h at 4 °C on ice, and then incubated for 30 min with 16 nM GPC-1-ADC (clone 01a033). The cells were then partitioned into two groups. For one group, internalisation was assessed upon incubation at 37 °C (100 µL vial^−1^). The other group served as a control for total cell surface binding, and was incubated at 4 °C. After the indicated incubation times, the cells were washed thrice with ice-cold PBS–0.2% (w/v) BSA buffer. The cells were incubated with 1 µg mL^–1^ biotinylated mouse anti-GPC-1 antibody (clone 02b006) for 30 min at 4 °C. The remaining surface expression was visualised after quenching with 50 µL Streptavidin-PE (BD Pharmingen, San Diego, CA, USA) for 30 min at 4 °C. Fluorescence intensities were determined with a FACS Canto II cytometer (Becton Dickinson, Mountain View, CA, USA) and recorded as the median fluorescence intensities (MFI). Internalisation was quantified by calculating the % internalisation as follows: MFI of the cell surface GPC-1 after induction of internalisation as detected by anti-GPC-1 mAb (clone 02b006) divided by the total bound anti-GPC-1 mAb (clone 02b006) multiplied by 100 and subtracted from 100.

### Fluorescence microscopy

An 18-mm microcover glass (Matsunami Glass Ind., Osaka, Japan) was set in a 12-well plate. BxPC-3 cells were seeded at a density of 2.5 × 10^4^ cells well^−1^ and incubated for 48 h. After 1-h incubation at 4 °C, the cells were incubated with 10 µg mL^–1^ GPC-1-ADC for 30 min at 4 °C followed by 2 h at 37 °C. Unbound antibody was then washed off with PBS, and the cells were fixed in 100% methanol for 15 min at −30 °C. The cells were then permeabilised. Nonspecific labelling was blocked in PBS + 0.3% (w/v) Triton X-100 + 1% (w/v) bovine serum albumin (BSA) for 1 h at room temperature. Plasma membrane and intracellular GPC-1-ADC were visualised by incubating the cells with Alexa Fluor 488-labelled donkey anti-mouse IgG (Life Technologies Corp., Carlsbad, CA, USA). Lysosomes were visualised by staining with an antibody directed against lysosome-associated membrane protein 1 (LAMP-1, Cell Signaling Technologies, Danvers, MA, USA) and the secondary antibody Alexa Fluor 647-labelled donkey anti-rabbit IgG (Life Technologies Corp., Carlsbad, CA, USA). Nuclei were visualised with 4′,6-diamidino-2-phenylindole (DAPI, Vector Laboratories, Burlingame, CA, USA). Images were acquired with a confocal microscope (LMS710; Carl Zeiss AG, Oberkochen, Germany).

### Establishment of GPC-1-knockdown cells

To generate a stable GPC-1-knockdown cell line, BxPC-3 cells were transfected with a commercial plasmid vector expressing short-hairpin RNA (shRNA) targeting GPC-1 mRNA or a negative nonspecific shRNA control (SuperArray Bioscience Corp., Frederick, MD, USA) using Lipofectamine^TM^ 2000 (Life Technologies Corp., Carlsbad, CA, USA). Transfected cells were selected using 600 µg mL^−1^ G418 (Life Technologies Corp., Carlsbad, CA, USA) and maintained in 250 µg mL^−1^ G418. A GPC-1-knockdown cell line was established and designated BxPC-3 KD-2-23. A BxPC-3 control cell line was established and designated BxPC-3 NC-11 via stable transfection with an empty vector. GPC-1 knockdown of transfected cells was assessed by fluorescence-activated cell sorting (FACS) and quantitative reverse transcription-PCR (qRT-PCR).

### Quantitative reverse transcription-PCR (qRT-PCR) analysis

BxPC-3, BxPC-3 NC-11 and BxPC-3 KD-2-23 cells were cultured in six-well plates at a density of 4.0 × 10^5^ cells well^−1^. After 24 h, total RNA was extracted and purified using RNeasy Mini Kit (QIAGEN, Valencia, CA), and cDNA was prepared using QuantiTect Reverse Transcription Kits (QIAGEN). To confirm the expression of GPC-1, qRT-PCR was performed as previously described.^25^ β-actin was used as a housekeeping gene for quantitative real-time PCR normalisation. Primer sequences used were as follows: GPC-1, forward primer 5′-GCCAGATCTACGGAGCCAAG-3′ and reverse primer 5′-AGGTTCTCCTCCATCTCGCT-3′ and β-actin, forward primer 5′-GTGGGGCGCCCCAGGCACCA-3′ and reverse primer 5′- CTCCTTAATGTCACGCACGATTTC-3′.

### In vivo efficacy study in pancreatic cancer cell line xenograft model

In this study, since it is essential to provide the efficacy and safety of this drug in animal models, we carried out animal experiments basically based on the previous report.^23^ Healthy female CB17/severe combined immunodeficient (SCID) mice aged 6 weeks were obtained from Charles River Japan (Yokohama, Japan). The animals were maintained in a pathogen-free facility in the National Institute of Biomedical Innovation, Health and Nutrition. Mice were housed in a temperature-controlled room with a 12-h light/12-h dark cycle, and provided free access to water. For the xenograft experiments, the mice were anaesthetised by 3% isoflurane, and subcutaneously injected in the flank with 5 × 10^6^ BxPC-3 cells in 100 μL of 1:1 (v/v) PBS:Matrigel (BD Biosciences, Franklin Lakes, NJ, USA). When the tumour volumes were > 100 mm^3^, the mice were randomly divided into five groups (8–9 per group). PBS, control ADC (10 mg kg^−1^) or GPC-1-ADC (1 mg kg^−1^, 3 mg kg^−1^ or 10 mg kg^−1^) was injected from caudal veins every 4 days until four doses had been administered. Tumour sizes were measured every 4 days using a vernier calliper. Tumour volumes were calculated as W^2^ × L/2, where W = width = smaller dimension and L = length = larger dimension. Body weights of mice were measured every 4 days. Mice were anaesthetised by 3% isoflurane and euthanised via cervical dislocation 36 days after the first treatment. Then, tumours were resected and weighed.

To investigate the pharmacologic action of GPC-1-ADC at the cellular level, animals with the BxPC-3 tumour xenograft were injected with PBS, control ADC (10 mg kg^−1^) or GPC-1-ADC (1 mg kg^−1^, 3 mg kg^−1^ or 10 mg kg^−1^), and the tumours were harvested 24 h later. Tumours were fixed in formalin, embedded in paraffin and sliced into 3-μm sections. IHC was performed using anti-phospho-histone H3 (Ser10) (#9701, 1:400, Cell Signaling Technologies, Danvers, MA, USA).

### In vivo efficacy study in patient-derived xenograft model

The use of human tissues was permitted by the Ethics Committees of the Osaka University Graduate School of Medicine and the National Institute of Biomedical Innovation, Health and Nutrition. Surgically resected samples from a patient who received no preoperative radiation or chemotherapy were cut into 3–4-mm pieces and subcutaneously transplanted into 6-week female NOD/Shi-scid-IL2Rγ null (NOG) mice. Mice were housed in a temperature-controlled room with a 12-h light/12-h dark cycle and provided free access to water. Mice were observed daily for tumour growth. The tumours were passaged once or twice. Mice were anaesthetised by 3% isoflurane, when the tumours were transplanted or passaged.

When the tumour volumes reached >100 mm^3^, the mice were randomly divided into five groups (six per group). PBS, control ADC (10 mg kg^−1^) or GPC-1-ADC (1 mg kg^−1^, 3 mg kg^−1^ or 10 mg kg^−1^) was injected from caudal veins every 4 days until four doses had been administered. Tumour sizes and body weights of mice were measured every 4 days. Tumour volumes were calculated as described above. Mice were euthanised 28 days after the first treatment, and tumours were resected and weighed. IHC of anti-phospho-histone H3 (Ser10) was performed as described above. All animal experiments were approved by the Institutional Review Board of the National Institute of Biomedical Innovation, Health and Nutrition (No. DS25-39R1).

### Statistical analyses

Overall survival (OS) and disease-free survival (DFS) were evaluated using the Kaplan–Meier method, and assessed by the log-rank test. Data are means ± SD for in vitro experiments and means ± SEM for in vivo experiments. To test for statistically significant differences between pairs of treatment means, one-way ANOVA was used followed by Holm’s post hoc test. Differences were considered significant at *P* < 0.05. All analyses were performed using JMP version 13.0 (SAS Institute, Cary, NC).

## Results

### GPC-1 expression in pancreatic cancer specimens

**Fig. 1:**
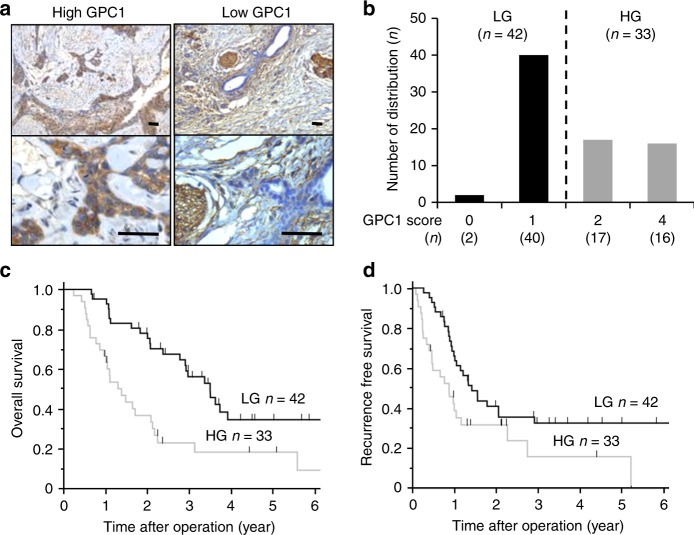
GPC-1 expression in clinical pancreatic cancer specimens. **a** Representative images of IHC staining for GPC in pancreatic cancer specimens. GPC-1 expression in cell membranes of human pancreatic cancer. Left panel: the high GPC-1 expression group (HG); right panel: the low GPC-1 expression group (LG). Scale bar: 50 μm. **b** The distribution of GPC-1 scores in LG and HG. **c** Kaplan–Meier analyses of OS. The black line represents LG and the grey line represents HG. **d** Kaplan–Meier analyses of RFS.

Among the 75 cases, we divided them into 2 equally balanced groups by GPC-1-stained score. Thirty-three cases (44%) scored more than 2 points (denoted as our high-expression group (HG), Fig. 1a, left panel). The remaining 42 cases (56%) scored less than 1 point (low-expression group (LG), Fig. 1a, right panel). The distribution of GPC-1 expression is shown in Fig. 1b.

We analysed the relation between GPC-1 expression in pancreatic cancer and various clinicopathological parameters, and found the correlation between high expression of GPC-1 and lymph node metastasis (Supplementary Table 1). Furthermore, GPC-1 expression significantly correlated with poor OS (*P* = 0.004, log-rank test) (Fig. 1c) and RFS (*P* = 0.021, log-rank test) (Fig. 1d).

### GPC-1 expression in human pancreatic cancer cell lines

**Fig. 2:**
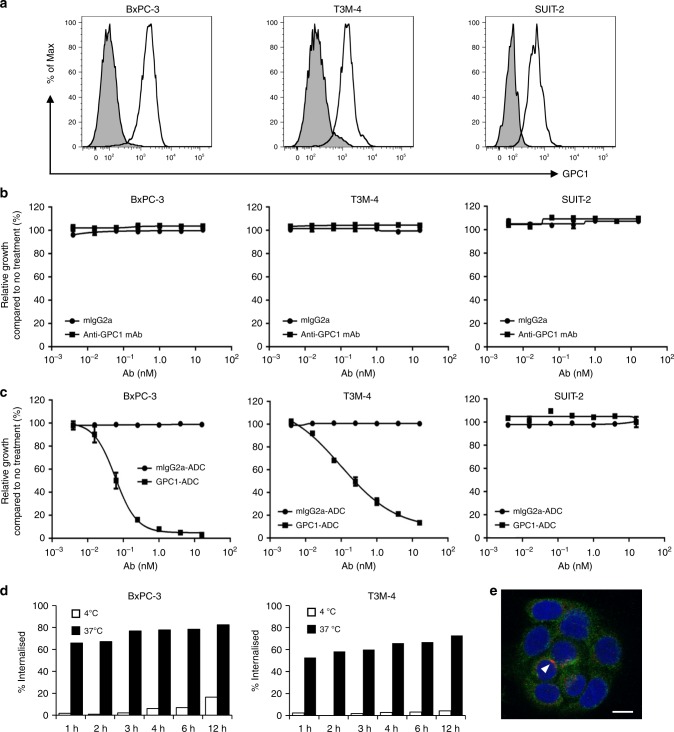
In vitro cell growth inhibition by ADC and internalisation of GPC-1 ADC. **a** Flow cytometry of GPC-1 expression in BxPC-3, T3M-4 and SUIT-2 using anti-GPC-1 monoclonal antibody. **b** BxPC-3, T3M-4 and SUIT-2 cells were treated with anti-GPC-1 monoclonal antibody (clone 01a033) or control IgG antibody for 144 h. Neither antibody inhibited the growth of any cell line. **c** Cells were treated with GPC-1-ADC or mouse IgG2a-ADC (a control ADC) for 144 h. Relative to control ADC, GPC-1-ADC significantly inhibited the growth of GPC-1-positive BxPC-3 and T3M-4 cell lines. Neither treatment inhibited the growth of the GPC-1-negative SUIT-2 cell line. **d** Time course of the internalisation activity of GPC-1-ADC in BxPC-3 and T3M-4 cells. **e** GPC-1-ADC internalises and locates in the lysosomes of BxPC-3 cells. Plasma membrane and intracellular GPC-1 were visualised by confocal fluorescence microscopy. Green indicates GPC-1-ADC, red indicates the lysosomal marker LAMP-1 and blue indicates DAPI (4′,6-diamidino-2-phenylindole)-stained DNA. Scale bar: 10 μm. GPC-1-ADC was found in the lysosomes. GPC-1-ADC and the lysosomal marker LAMP-1 overlapped (arrow).

Flow cytometry was performed to measure GPC-1 protein on pancreatic cancer cell surfaces. GPC-1 expression was high in BxPC-3 and T3M-4 and low in SUIT-2 (Fig. 2a). Quantitation of GPC-1 expression on the plasma membrane by indirect immunofluorescence assay indicated that it was upregulated in BxPC-3 (93290 sites cell^−1^) and T3M-4 (76850 sites cell^−1^) but expressed at low levels in SUIT-2 (25444 sites cell^−1^) (Table 1).

**Table 1:** IC_50_ of MMAF and anti-glypican-1 ADC in human pancreatic cancer cell lines.

Cell lines	Glypican-1 expression (ABC cell^−1^)	MMAF (nM)	MMAF in anti-glypican-1 ADC (nM)
BxPC-3	93290	31.3	0.063
T3M-4	76850	24.4	0.24
SUIT-2	25444	459.5	N/A

*ABC* antibody-binding capacity.

### Cytotoxicity studies with GPC-1-ADC

A cell growth assay with ADCs was performed using the GPC-1-positive pancreatic cancer cell lines BxPC-3 and T3M-4. SUIT-2 served as a negative control. Unconjugated anti-GPC-1 mAb had no effect on the viability of any cell line (Fig. 2b). Nevertheless, GPC-1-ADC caused a dose-dependent decrease in the viability of BxPC-3 and T3M-4 in vitro (Fig. 2c). The IC_50_ values of GPC-1-ADC were 0.063 nM for BxPC-3 and 0.24 nM for T3M-4, respectively. However, GPC-1-ADC had little effect on SUIT-2 cells (Fig. 2c). The IC_50_ of GPC-1-ADC was not calculated for SUIT-2 as the 16-nM cell inhibitory rate of GPC-1-ADC did not reach 50% (Fig. 2c). Unconjugated GPC-1 mAb was not cytotoxic at concentrations ≤ 666.6 nM (data not shown). As MMAF impairs plasma membrane permeability, MMAF sensitivity was low. The IC_50_ values for MMAF against the cell lines were in the range of 24.4–459.5 nM (Table 1).

### Internalisation of GPC-1-ADC

The binding capacity and percentage of internalisation of GPC-1-ADC were determined for BxPC-3 and T3M-4 by flow cytometry. Residual cell surface GPC-1 was measured after each GPC-1-ADC exposure time point using biotinylated anti-GPC-1 mAb (clone 02b006). GPC-1-ADC internalisation occurred rapidly in both cell lines (Fig. 2d). An immunofluorescence analysis was conducted to confirm GPC-1-ADC translocation to the lysosomes. GPC-1-ADC bound to the membranes of cells preincubated at 4 °C. When BxPC-3 exposed to GPC-1-ADC was incubated at 37 °C for 2 h, GPC-1-ADC appeared in the lysosomes. It overlapped with the lysosomal marker LAMP-1 (Fig. 2e). Thus, GPC-1-ADC first binds to the GPC-1 on the membranes of GPC-1-expressing cells, is internalised and then translocates to the lysosomes.

### Cytotoxicity studies with GPC-1-knockdown cell line

**Fig. 3:**
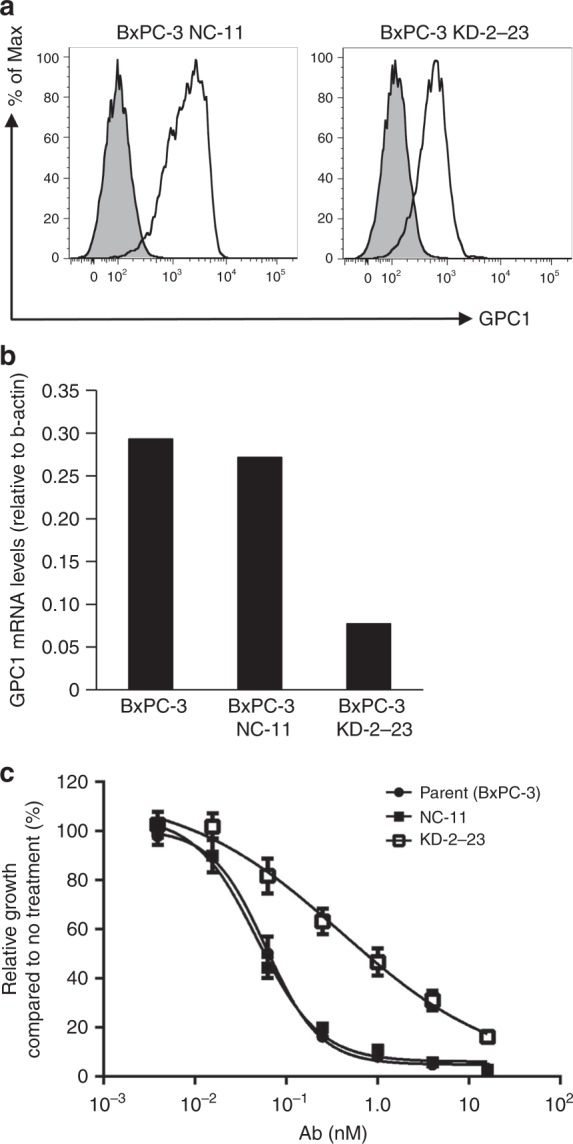
Cytotoxicity studies with GPC-1 knockdown cell line. **a** Flow cytometry of GPC-1 expression in a GPC-1-knockdown cell line (BxPC-3 KD-2-23) and a control cell line (BxPC-3 NC-11) using anti-GPC-1 monoclonal antibody. **b** Quantitative reverse transcription-PCR analysis of GPC-1 mRNA levels relative to β-actin in a GPC-1-knockdown cell line (BxPC-3 KD-2-23) and control cell lines (BxPC-3 and BxPC-3 NC-11). **c** BxPC-3, BxPC-3 NC-11 and BxPC-3 KD-2-23 were treated with GPC-1-ADC for 144 h. GPC-1-ADC had a lower growth inhibition effect of the GPC-1-knockdown cell line (BxPC-3 KD-2-23) than the parent BxPC-3 and the negative control cell line (BxPC-3 NC-11).

We investigated the association between GPC-1 expression and GPC-1-ADC cytotoxicity using GPC-1-knockdown BxPC-3. Both BxPC-3 and BxPC-3 NC-11 (negative control cell line) expressed GPC-1, whereas the GPC-1-knockdown BxPC-3 KD-2-23 was GPC-1-negative according to flow cytometry (Fig. 3a) and qRT-PCR (Fig. 3b). We performed a cell growth assay, and confirmed that GPC-1-ADC reduced BxPC-3 and BxPC-3 NC-11 viability in a dose-dependent manner. In contrast, GPC-1-ADC had little effect on BxPC-3 KD-2-23 (Fig. 3c, Supplementary Table 2).

### In vivo efficacy study in BxPC-3 xenograft

**Fig. 4:**
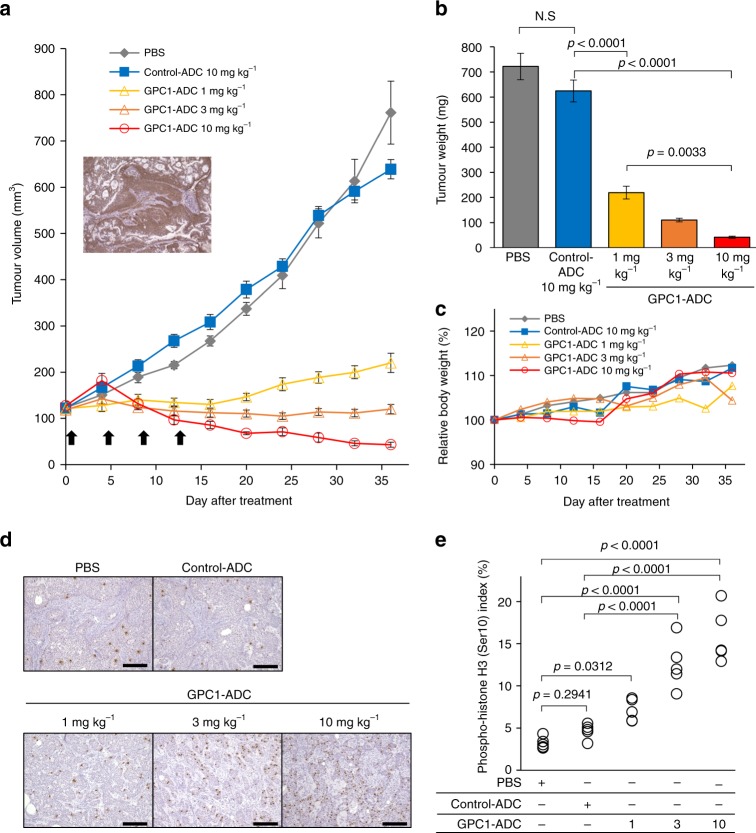
Antitumour activity of GPC-1-ADC in BxPC-3 xenograft. **a** Antitumour efficacy of GPC-1-ADC in BxPC-3 xenograft models (*n* = 8 or 9). Representative images of IHC staining for GPC-1 in xenografted tumour tissues from untreated mice. Tumour-bearing mice were intravenously administered PBS, control ADC (10 mg kg^–1^) or GPC-1-ADC (1 mg kg^–1^, 3 mg kg^–1^ or 10 mg kg^–1^) on days 0, 4, 8 and 12. Each point on the graph represents the average tumour volume. **b** Thirty-six days after the first treatment, the tumour weights were calculated. **c**. Changes in body weight are represented. **d** GPC-1-ADC causes mitotic arrest in vivo. Animals with BxPC-3 tumour xenografts were administered a single dose of PBS, control ADC (10 mg kg^–1^) or GPC-1-ADC (1 mg kg^–1^, 3 mg kg^–1^ or 10 mg kg^–1^). After 24 h, the tumours were harvested and stained with anti-phospho-histone H3 (Ser10) antibody to detect mitotic cells. Scale bar: 200 μm. **e**. Phospho-histone H3 (Ser10) staining was recorded as the ratio of positively stained cells to all tumour cells in five fields (×200 magnification).

SCID mice were subcutaneously inoculated with BxPC-3 cells, and then intravenously treated with 1 mg kg^–1^, 3 mg kg^–1^ or 10 mg kg^–1^ GPC-1-ADC once every 4 days for a total of four doses (Fig. 4a). GPC-1 expression in the BxPC-3 xenograft was confirmed by immunohistochemistry (IHC) (Fig. 4a). Compared with PBS and control ADC, GPC-1-ADC administration significantly inhibited BxPC-3 xenograft growth as assessed by tumour volume and weight (Fig. 4a, b). Tumour volume was significantly decreased by 10 mg kg^–1^ GPC-1-ADC. No significant weight loss was observed in any group (Fig. 4c). The BxPC-3 xenograft tumours were stained with phosphorylated histone H3 (Ser10), which is related to chromosome condensation. The aim was to analyse the pharmacologic action of GPC-1-ADC in vivo using a mitotic marker antibody. A dramatic increase in the percentage of mitotically active tumour cells was observed following GPC-1-ADC treatment but not in the control ADC (Fig. 4d, e). Therefore, the tubulin-polymerising inhibitor MMAF was successfully delivered to the GPC-1-expressing tumour cells via anti-GPC-1 mAb and caused mitotic arrest.

### In vivo efficacy study in patient-derived xenograft

**Fig. 5:**
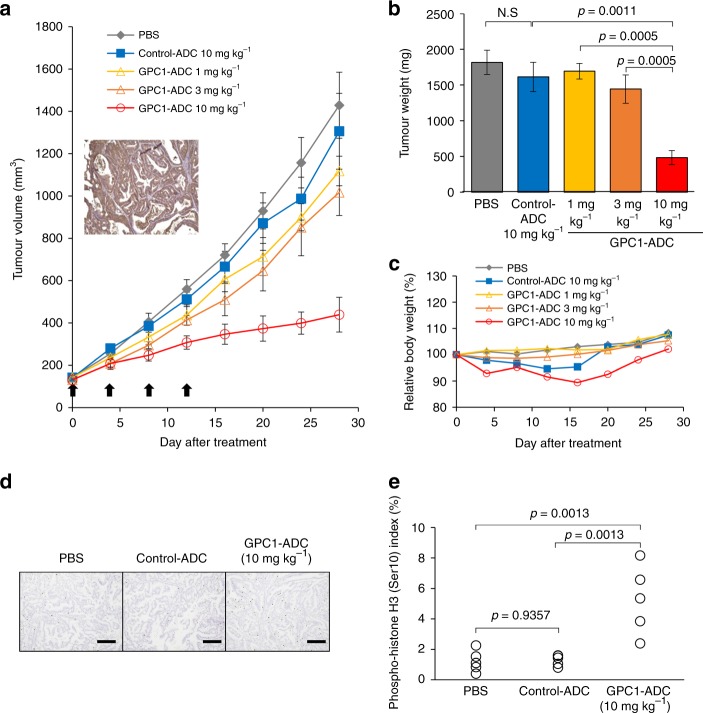
Antitumour activity of GPC-1-ADC in patient-derived xenograft. **a** Antitumour efficacy of GPC-1-ADC in patient-derived xenograft models (*n* = 6). Representative images of IHC staining for GPC-1 in xenografted tumour tissues from untreated mice. Tumour-bearing mice were intravenously administered PBS, control ADC (10 mg kg^–1^) or GPC-1-ADC (1 mg kg^–1^, 3 mg kg^–1^ or 10 mg kg^–1^) on days 0, 4, 8 and 12. Each point on the graph represents the average tumour volume. **b** Twenty-eight days after the first treatment, the tumour weights were calculated. **c** Changes in body weight are represented. **d** GPC-1-ADC causes mitotic arrest in vivo. Animals with patient-derived xenografts were administered a single dose of PBS, control ADC (10 mg kg^–1^) or GPC-1-ADC (10 mg kg^–1^). After 24 h, tumours were harvested and stained with anti-phospho-histone H3 (Ser10) antibody to detect mitotic cells. Scale bar: 200 μm. **e** Phospho-histone H3 (Ser10) staining was recorded as the ratio of positively stained cells to all tumour cells in five fields (×200 magnification).

We also assessed the antitumour efficacy of GPC-1-ADC against a pancreatic cancer patient tumour-derived xenograft (PDX). Pancreatic cancer tissues were subcutaneously implanted in NOG mice. The animals then received intravenous 1 mg kg^–1^, 3 mg kg^–1^ or 10 mg kg^–1^ GPC-1-ADC every 4 days for a total of four doses (Fig. 5a). GPC-1 expression in the tumour tissue was confirmed by IHC (Fig. 5a). PDX tumour growth in the 10 mg kg^–1^ GPC-1-ADC group was significantly suppressed relative to the control-ADC group (Fig. 5a, b). No significant weight loss was observed in any group (Fig. 5c). The PDX tumours were stained with phosphorylated histone H3 (Ser10). A substantial increase in the percentage of mitotic tumour cells was detected following GPC-1-ADC treatment but not in response to the control ADC (Fig. 5d, e).

## Discussion

We previously identified glypican-1 (GPC-1) by quantitative proteomics as an antigen for oesophageal squamous cell carcinoma (ESCC). We focused on the cell membrane protein and confirmed relatively low GPC-1 expression in normal tissues relative to ESCC.^19^ GPC-1 expression in ESCC is related to poor prognosis and chemoresistance, as previously reported.^19^ In this study, IHC analyses showed that 97.3% of PDAC specimens were GPC-1 positive, and GPC-1 overexpression was related to poor prognosis. We demonstrated that targeting GPC-1 with anti-GPC-1 mAb (clone 1–12) had a strong antitumour effect via ADCC and CDC in a GPC-1-positive ESCC xenograft model.^19,20^ We showed the efficacy of anti-GPC-1 mAb after confirming GPC-1 expression in PDAC. However, we could not prove that it had the same efficacy as ESCC (Supplementary Fig. 1). GPC-1 expression may be weaker in pancreatic than oesophageal cancer, and its roles may differ in each case.

We reported the establishment of a new ADC-based system combining anti-GPC-1 mAb with MMAF. The latter inhibits mitosis by suppressing tubulin polymerisation and disrupting the microtubule network in proliferating cells. It has shown remarkable efficacy against uterine cervical cancer.^23^ ADC efficacy depends mainly on target antigen expression on tumour cells, ADC-binding affinity to the antigen, cellular internalisation and conjugated drug potency. Our in vitro ADC assay showed that cancer cells highly expressing GPC-1 (namely BxPC-3 and T3M-4) were highly sensitive to GPC-1-ADC, whilst those expressing GPC-1 only at low levels (such as SUIT-2) were comparatively resistant to it. Furthermore, the GPC-1-knockdown cell (KD-2-23) derived from GPC-1-positive BxPC-3 was also relatively insensitive to GPC-1-ADC. Thus, GPC-1 expression is necessary to enable GPC-1-ADC to inhibit cancer cell growth. Moreover, anti-GPC-1 mAb was rapidly internalised into the cancer cells after GPC-1 bound to them. Therefore, GPC-1 is a suitable target for ADC. GPC-1 expression was very weak or undetectable in various normal cells.^20^ For this reason, specific efficacy of GPC-1-ADC to PDAC is expected. Since there are some reports that GPC-1 is reported to be shed into the extracellular matrix, and high GPC-1-expression tumours secrete a lot of GPC-1 and lead to high serum GPC-1 level,^21,26^ we might predict an effect of GPC-1-ADC by measuring the serum GPC-1 levels.

To formulate GPC-1-ADC, we conjugated MMAF with anti-GPC-1 mAb. MMAF inhibits mitosis by suppressing tubulin polymerisation and disrupting the microtubule network in proliferating cells. MMAF is a novel auristatin derivative with a charged *C*-terminal phenylalanine that attenuates its cytotoxicity compared with its uncharged counterpart monomethyl auristatin E (MMAE).^24,27–29^ The carboxylic acid terminus of free MMAF limits its passive transit through cell membranes.^24,30,31^ MMAF has potent antitumour effects when it is conjugated via a protease-cleavable linker to a monoclonal antibody that targets internalised tumour-specific cell surface antigens. The linker to the monoclonal antibody is stable in extracellular fluid, but the conjugate is cleaved by cathepsin B upon entry into the tumour cells. Thence, it activates the antimitotic mechanism.^29^

GPC-1-ADC had a significant antitumour effect in BxPC-3-xenografted and PDX mice. However, the response was stronger in the former than the latter. GPC-1 expression was heterogeneous in the PDX mice, but homogeneous in the BxPC-3-xenografted mice according to IHC. Therefore, these two models differed in terms of GPC-1-ADC sensitivity. Moreover, as the antitumour effect of MMAF differs among PDAC cell lines (Table 1), a safer and more effective antibody–drug must be sought for clinical applications. We previously reported that GPC-1 expressions in normal tissues and organs are very low compared with cancer tissues.^20^ In addition, since anti-GPC-1 mAb cross-reacts with mouse GPC-1, a preliminary safety test of GPC-1-ADC was conducted using male and female C57B/6 J mice. As a result, the histopathologic examination revealed only a small number of inflammatory cell infiltrates in the liver of the GPC-1-ADC group, and we confirmed that this therapeutic cytotoxic agent was tolerable at the curative dose.^23^ We also verified that no significant weight loss was observed in any group receiving GPC-1-ADC.

There were several limitations to this study. First, GPC-1 expression was heterogeneous in clinical pancreatic cancer. This discrepancy may account for the observed differences in GPC-1-ADC antitumour efficacy among the pancreatic cancer cell lines. Nevertheless, recent studies indicated that the chemistry of the drug may determine whether it can diffuse into the surrounding cells and cause ‘bystander killing’.^29,30^ Furthermore, the extent to which ADC mediates bystander killing depends largely on ADC internalisation after it binds to the target antigen, whether the linker is cleavable, and the hydrophobicity of the cytotoxic warhead. All of these factors must be established for the clinical application of GPC-1-ADC. Second, we must develop GPC-1-ADC using humanised anti-GPC-1 monoclonal antibody, and validate its clinical efficacy and safety. We selected an anti-GPC-1 antibody with strong internalisation capacity.^23^ GPC-1-ADC immediately internalised GPC-1-positive cancer cells. We may be able to develop more efficacious GPC-1-ADC using antibodies with high internalised activity.

The GPC-1-ADC developed in this study showed significant tumour growth inhibition against GPC-1-positive pancreatic cells and patient-derived, GPC-1-positive pancreatic cancers. Our preclinical data demonstrated that targeting GPC-1 by ADC is a promising therapy for patients with GPC-1-positive pancreatic cancer.

## Supplementary information

Supplementary Imformation

## Data Availability

All data generated or analysed during this study are included in this published article and its supplementary information files.

## References

[1] Rahib L, Smith BD, Aizenberg R, Rosenzweig AB, Fleshman JM, Matrisian LM (2014). Projecting cancer incidence and deaths to 2030: the unexpected burden of thyroid, liver, and pancreas cancers in the United States. Cancer Res..

[2] Uesaka K, Boku N, Fukutomi A, Okamura Y, Konishi M, Matsumoto I (2016). Adjuvant chemotherapy of S-1 versus gemcitabine for resected pancreatic cancer: a phase 3, open-label, randomised, non-inferiority trial (JASPAC 01). Lancet.

[3] Conroy T, Hammel P, Hebbar M, Ben Abdelghani M, Wei AC, Raoul JL (2018). FOLFIRINOX or gemcitabine as adjuvant therapy for pancreatic cancer. N. Engl. J. Med..

[4] Hartwig W, Werner J, Jager D, Debus J, Buchler MW (2013). Improvement of surgical results for pancreatic cancer. Lancet Oncol..

[5] Siegel RL, Miller KD, Jemal A (2016). Cancer statistics, 2016. CA Cancer J. Clin..

[6] Von Hoff DD, Ervin T, Arena FP, Chiorean EG, Infante J, Moore M (2013). Increased survival in pancreatic cancer with nab-paclitaxel plus gemcitabine. N. Engl. J. Med..

[7] Conroy T, Desseigne F, Ychou M, Bouche O, Guimbaud R, Becouarn Y (2011). FOLFIRINOX versus gemcitabine for metastatic pancreatic cancer. N. Engl. J. Med..

[8] Sievers EL, Senter PD (2013). Antibody-drug conjugates in cancer therapy. Annu Rev. Med..

[9] Polakis P (2016). Antibody Drug Conjugates for Cancer Therapy. Pharm. Rev..

[10] Katz J, Janik JE, Younes A (2011). Brentuximab Vedotin (SGN-35). Clin. Cancer Res..

[11] Younes A, Gopal AK, Smith SE, Ansell SM, Rosenblatt JD, Savage KJ (2012). Results of a pivotal phase II study of brentuximab vedotin for patients with relapsed or refractory Hodgkin’s lymphoma. J. Clin. Oncol..

[12] Burris HA, Rugo HS, Vukelja SJ, Vogel CL, Borson RA, Limentani S (2011). Phase II study of the antibody drug conjugate trastuzumab-DM1 for the treatment of human epidermal growth factor receptor 2 (HER2)-positive breast cancer after prior HER2-directed therapy. J. Clin. Oncol..

[13] LoRusso PM, Weiss D, Guardino E, Girish S, Sliwkowski MX (2011). Trastuzumab emtansine: a unique antibody-drug conjugate in development for human epidermal growth factor receptor 2-positive cancer. Clin. Cancer Res..

[14] Krop IE, LoRusso P, Miller KD, Modi S, Yardley D, Rodriguez G (2012). A phase II study of trastuzumab emtansine in patients with human epidermal growth factor receptor 2-positive metastatic breast cancer who were previously treated with trastuzumab, lapatinib, an anthracycline, a taxane, and capecitabine. J. Clin. Oncol..

[15] Goy A, Forero A, Wagner-Johnston N, Christopher Ehmann W, Tsai M, Hatake K (2016). A phase 2 study of inotuzumab ozogamicin in patients with indolent B-cell non-Hodgkin lymphoma refractory to rituximab alone, rituximab and chemotherapy, or radioimmunotherapy. Br. J. Haematol..

[16] Ogura M, Tobinai K, Hatake K, Davies A, Crump M, Ananthakrishnan R (2016). Phase I Study of Inotuzumab Ozogamicin Combined with R-CVP for Relapsed/Refractory CD22+ B-cell Non-Hodgkin Lymphoma. Clin. Cancer Res..

[17] Filmus J, Selleck SB (2001). Glypicans: proteoglycans with a surprise. J. Clin. Invest..

[18] Filmus J, Capurro M, Rast J (2008). Glypicans. Genome Biol..

[19] Hara H, Takahashi T, Serada S, Fujimoto M, Ohkawara T, Nakatsuka R (2016). Overexpression of glypican-1 implicates poor prognosis and their chemoresistance in oesophageal squamous cell carcinoma. Br. J. Cancer.

[20] Harada E, Serada S, Fujimoto M, Takahashi Y, Takahashi T, Hara H (2017). Glypican-1 targeted antibody-based therapy induces preclinical antitumor activity against esophageal squamous cell carcinoma. Oncotarget.

[21] Melo SA, Luecke LB, Kahlert C, Fernandez AF, Gammon ST, Kaye J (2015). Glypican-1 identifies cancer exosomes and detects early pancreatic cancer. Nature.

[22] Kleeff J, Ishiwata T, Kumbasar A, Friess H, Büchler MW, Lander AD (1998). The cell-surface heparan sulfate proteoglycan glypican-1 regulates growth factor action in pancreatic carcinoma cells and is overexpressed in human pancreatic cancer. J. Clin. Invest..

[23] Matsuzaki S, Serada S, Hiramatsu K, Nojima S, Matsuzaki S, Ueda Y (2018). Anti-glypican-1 antibody-drug conjugate exhibits potent preclinical antitumor activity against glypican-1 positive uterine cervical cancer. Int J. Cancer.

[24] Doronina SO, Mendelsohn BA, Bovee TD, Cerveny CG, Alley SC, Meyer DL (2006). Enhanced activity of monomethylauristatin F through monoclonal antibody delivery: effects of linker technology on efficacy and toxicity. Bioconjug Chem..

[25] Yokoyama T, Enomoto T, Serada S, Morimoto A, Matsuzaki S, Ueda Y (2013). Plasma membrane proteomics identifies bone marrow stromal antigen 2 as a potential therapeutic target in endometrial cancer. Int J. Cancer.

[26] Zhou CY, Dong YP, Sun X, Sui X, Zhu H, Zhao YQ (2018). High levels of serum glypican-1 indicate poor prognosis in pancreatic ductal adenocarcinoma. Cancer Med..

[27] Smith LM, Nesterova A, Alley SC, Torgov MY, Carter PJ (2006). Potent cytotoxicity of an auristatin-containing antibody-drug conjugate targeting melanoma cells expressing melanotransferrin/p97. Mol. Cancer Ther..

[28] Oflazoglu E, Stone IJ, Gordon K, Wood CG, Repasky EA, Grewal IS (2008). Potent anticarcinoma activity of the humanized anti-CD70 antibody h1F6 conjugated to the tubulin inhibitor auristatin via an uncleavable linker. Clin. Cancer Res..

[29] Nilsson R, Mårtensson L, Eriksson SE, Sjögren HO, Tennvall J (2010). Toxicity-reducing potential of extracorporeal affinity adsorption treatment in combination with the auristatin-conjugated monoclonal antibody BR96 in a syngeneic rat tumor model. Cancer.

[30] Ogitani Y, Hagihara K, Oitate M, Naito H, Agatsuma T (2016). Bystander killing effect of DS-8201a, a novel anti-human epidermal growth factor receptor 2 antibody-drug conjugate, in tumors with human epidermal growth factor receptor 2 heterogeneity. Cancer Sci..

[31] Staudacher AH, Brown MP (2017). Antibody drug conjugates and bystander killing: is antigen-dependent internalisation required?. Br. J. Cancer.

