# Supplementary material for: Anti-glypican-1 antibody–drug conjugate is a potential therapy against pancreatic cancer
Source: Br J Cancer. 2020 Mar 10;122(9):1333–41. doi: 10.1038/s41416-020-0781-2 (PMC7189381; doi:10.1038/s41416-020-0781-2)
Supplement: Supplementary file 1 — Supplementary Imformation [file 41416_2020_781_MOESM1_ESM.pdf]

Supplementary figure 1

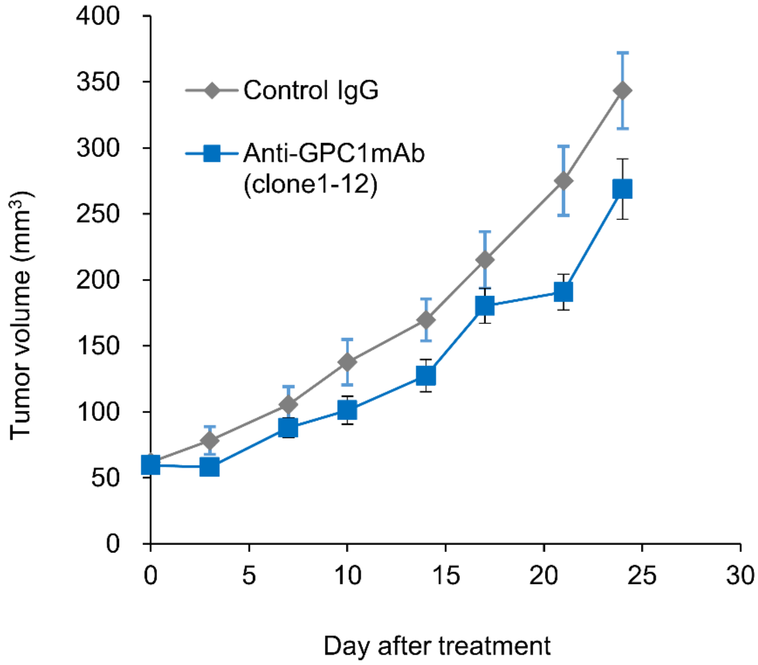

Supplementary Figure 1. Antitumour activity of anti-GPC1 mAb in BxPC-3 xenograft

Antitumour efficacy of anti-GPC1 mAb (clone 1-12) in BxPC-3 xenograft models (n = 10). Tumour-bearing mice were administered intraperitoneal control mAb (10 mg kg^{-1}) or anti-GPC1 mAb (10 mg kg^{-1}) as previously reported.[20] Each point on the graph represents the average tumour volume. Efficacy was not demonstrated

Supplementary Table 1.

Correlation between GPC1 expression and clinicopathological factors in our patient cohort.

	GPC1 expression		
Variables	Low (n=42)	High (n=33)	*p*-value
Gender (n)			0.20
Male	29	18	
Female	13	15	
Age (yrs.)			
Median	67.2	67.1	0.12
Diabetes mellitus (n)			0.16
yes	9	18	
no	24	24	
Histology type (n)			0.43
wel/mod	39	32	
por/muc	3	1	
Tumor location (n)			0.26
Pancreas head	24	23	
Pancreas body or tail	18	10	
pT stage (n)			0.06
T1-2	16	6	
T3-4	26	27	
pN stage (n)			0.048
N0	32	18	
N1	10	15	

Supplementary Table 2. IC_{50} of MMAF and anti-glypican-1 ADC in BxPC-3 and GPC1 knockdown cell line

Cell lines	Glypican-1 expression (ABC $cell^{-1}$)	MMAF (nM)	MMAF in anti-glypican-1 ADC (nM)
BxPC-3	93 290	31.3	0.063
BxPC-3 NC-11	83 880	26.8	0.053
BxPC-3 KD-2-23	18 946	24.4	0.76

ABC: antibody binding capacity
